# Freeing *Aspergillus fumigatus* of Polymycovirus Infection Renders It More Resistant to Competition with *Pseudomonas aeruginosa* Due to Altered Iron-Acquiring Tactics

**DOI:** 10.3390/jof7070497

**Published:** 2021-06-22

**Authors:** Rutuja H. Patil, Ioly Kotta-Loizou, Andrea Palyzová, Tomáš Pluháček, Robert H. A. Coutts, David A. Stevens, Vladimír Havlíček

**Affiliations:** 1 Institute of Microbiology of the Czech Academy of Sciences, Vídeňská 1083, 142 20 Prague, Czech Republic; rutuja.patil@biomed.cas.cz (R.H.P.); palyzova@biomed.cas.cz (A.P.); tomas.pluhacek@biomed.cas.cz (T.P.); vlhavlic@biomed.cas.cz (V.H.); 2 Department of Analytical Chemistry, Faculty of Science, Palacký University, 17. Listopadu 12, 771 46 Olomouc, Czech Republic; 3 Department of Life Sciences, Imperial College London, London SW7 2AZ, UK; i.kotta-loizou13@imperial.ac.uk; 4 Department of Clinical, Pharmaceutical and Biological Science, University of Hertfordshire, Hatfield AL10 9AB, UK; r.coutts@herts.ac.uk; 5 California Institute for Medical Research, 2260 Clove Dr., San Jose, CA 95128, USA; 6 Division of Infectious Diseases and Geographic Medicine, Stanford University School of Medicine, Stanford, CA 95128, USA

**Keywords:** *Aspergillus fumigatus*, intermicrobial competition, polymycovirus, *Pseudomonas aeruginosa*, siderophore

## Abstract

A virus-free (VF) *A. fumigatus* isolate has been shown to be resistant in competition with *Pseudomonas* as compared to the isogenic line infected with *Aspergillus fumigatus* polymycovirus 1 (AfuPmV-1), and this phenotype was apparently related to alterations in iron metabolism. Here we investigated further the mechanisms underpinning this phenotype. The extracellular siderophore profiles of five isogenic VF and virus-infected (VI) strains were sampled at 24, 31, 48, 54, and 72 h in submerged cultures and quantitatively examined by liquid chromatography and mass spectrometry. Intracellular profiles of conidia and cultures at the stationary growth phase were defined. VF *A. fumigatus* demonstrated the best fitness represented by the fastest onset of its exponential growth when grown on an iron-limited mineral medium. The exponential phase and transitional production phase of the extracellular triacetylfusarinine C (TafC) were achieved at 24 and 31 h, respectively, contrary to VI strains, which acted more slowly. As a result, the TafC reservoir was consumed sooner in the VF strain. Additionally, the VF strain had lower ferricrocin and higher hydroxyferricrocin content in the pellet during the stationary phase. All of these differences were significant (Kruskal–Wallis, *p* < 0.01). In our study, the siderophore reservoir of a VF strain was consumed sooner, improving the fitness of the VF strain in competition with *P. aeruginosa*.

## 1. Introduction

*Aspergillus* infections caused 600,000 deaths in 2019, making the opportunistic pathogens, *Aspergillus* spp., a global concern [1]. The high 2020 incidence of COVID-19-associated pulmonary aspergillosis has triggered a new quest for antifungal therapies [2]. Mycoviruses are widespread in all fungal taxa. Mycovirus families are diverse and can affect their fungal hosts, including inducing hypovirulence, hypervirulence, or a killer phenotype via toxin production [3]. However, the mechanisms controlling mycovirus-induced phenotypic alterations or host–virus protein–protein interactions are unclear. 

*Aspergillus fumigatus* and *Pseudomonas aeruginosa* cohabit and compete in nature, in both soil and water, and are commonly found as pathogens in the lungs of immunocompromised patients and persons with cystic fibrosis [4]. Aspergillus fumigatus polymycovirus 1 (AfuPmV-1) was initially discovered in the *A. fumigatus* UK AF293 isolate [5] and is the prototype member of the recently established family *Polymycoviridae*. A recent study showed that the virus-free (VF) *A. fumigatus* was more resistant in competition with *P. aeruginosa* as compared to the virus infected (VI) isogenic lines, with the mycovirus infection weakening *A. fumigatus* via altering fungal stress responses by a mechanism somehow linked to iron (Fe) metabolism [6]. The VF *A. fumigatus* was also significantly resistant to *P. aeruginosa* volatiles, small organic (probably lipophilic) molecules, possibly unrelated to Fe metabolism [7]. The latter finding may indicate that viral infection engenders a reduced host response to a variety of stress responses. In this communication, we report newly discovered iron-acquiring tactics used by VF *A. fumigatus* that make it more resistant in competition with *P. aeruginosa*. 

## 2. Materials and Methods

### 2.1. Isolates 

In a blinded study, five *A. fumigatus* strains (Table 1) [6] were maintained on malt extract agar (1.7% malt extract, 0.3% mycological peptone, 3% Bacto agar, pH 5.4; all chemicals from HiMedia, Czechia) for 10 days, at 37 °C. The *A. fumigatus* USA and UK AF293 strains were designated California Institute for Medical Research strain numbers 10–53 and 18–95. *A. fumigatus* UK AF293 strain (18–95) was cured of AfuPmV-1, using the protein synthesis inhibitor cycloheximide [5], producing a VF strain now designated 18–42. AfuPmV-1 was purified by differential polyethylene glycol precipitation and ultracentrifugation. Purified AfuPmV-1 was re-introduced in the VF *Aspergillus* by protoplast transfection, producing re-infected strains designated as 19–40 and 19–42. The presence or absence of AfuPmV-1 was confirmed by Northern blotting and RT-qPCR, as previously described [5].

### 2.2. Fungal Strain Cultivation 

For detection of metabolites, each strain was grown in an iron-limited mineral medium at pH 7 consisting of Na_2_HPO_4_·12H_2_O (14.62 g/L), KH_2_PO_4_ (3 g/L), NaCl (0.5 g/L), NH_4_Cl (1 g/L); source of carbon: glucose (5 g/L); trace elements: MgSO_4_∙7H_2_O (0.2 g/L), CaCl_2_·2H_2_O (0.05 g/L), ZnSO_4_·7H_2_O (0.01 g/L), MnSO_4_·H_2_O (0.017 g/L), CoCl_2_·6H_2_O (0.0048 g/L), CuSO_4_·5H_2_O (0.003 g/L), Na_2_MoO_4_ (0.0045 g/L). All chemicals were obtained from Lachner Chemicals (Neratovice, Czechia).

Conidia were harvested from one-week-old cultures grown at 37 °C on malt agar extract, using phosphate-buffered saline containing 0.1% Tween 80, and the suspension was filtered (5 µm SyringeStrainer, Pluriselect, San Diego, CA, USA). All cultures were inoculated with the same concentration of 10^8^ conidia per mL. The cultures were shaken for 72 h in flasks, at 37 °C, on an orbital shaker (190 rpm). All experiments were performed in three biological replicates. Supernatants containing conidia were separated from mycelia by filtration through a Whatman (VWR International, Stříbrná Skalice, Czechia) filter (0.2 μm), using a sterile funnel. The mycelia were washed three times with sterile water, followed by centrifugation at 14,000× *g*, 2 min, at room temperature. Optical microscopy was used to check for the presence of conidia in the filtrate and the absence of hyphal contamination.

### 2.3. Extraction of Siderophores and Calibration

For extraction of siderophores, two-step liquid-liquid extraction was performed. Briefly, mycelia were sonicated and centrifuged to release intracellular content. FeCl_3_ solution (100 μM) was added to the supernatant to saturate iron-free siderophores. Supernatants (50 or 100 µL for extracellular or intracellular metabolite screening, respectively) were spiked with ferrioxamine E (FoxE) as an internal standard at a final concentration of 100 or 50 ng/mL for extracellular or intracellular metabolites, respectively. The supernatants were extracted twice with ethyl acetate (triple volume) and dried under reduced pressure. The remaining aqueous phase was mixed with four volumes of methanol and frozen at −80 °C, 1 h. Precipitated proteins were removed by centrifugation (14,000× *g*, 4 °C, 10 min), and the supernatant layer was transferred to a vial with the evaporated ethyl acetate fraction and concentrated under reduced pressure. 

Standard triacetylfusarinine C (TafC), FoxE, and ferricrocin (FC) ferriforms were obtained from EMC Microcollections GmbH (Tübingen, Germany) and used for calibration curve construction. The hydroxyferricrocin (HFC) was quantified using the FC calibration curve, assuming the same ionization efficiency. The calibration sequence consisted of 0.5, 1, 5, 10, 50, 100, 500, and 1000 ng/mL of TafC and FC final siderophore concentrations in an HPLC vial. The limits of detection (LOD) and quantitation (LOQ) were defined as the lowest concentrations for which the standard deviation of the intercept equaled 3.3 and 10, respectively. All samples were analyzed in triplicate, and results expressed as means ± standard deviation. The instrument performance was checked by a system suitability test using the HPLC peptide standard mixture (Sigma-Aldrich, Prague, Czech Republic).

### 2.4. Liquid Chromatography and Mass Spectrometry

Pooled extracts were re-suspended in 5% LC–MS-grade acetonitrile (ACN; 100 µL and 5–50 mL for the screening of intracellular and extracellular metabolites, respectively) and injected onto an Acquity HSS T3 C18 analytical column (1.8 μm, 1.0 × 150 mm, Waters, Milford, MA, USA). Analytes were gradient-eluted with a 50 μL/min flow rate (A: 1% ACN with 0.1% formic acid in water; B: 95% ACN with 0.1% aqueous formic acid): 0 min, 2%; 2 min, 2%; 9 min, 60%; 11.0 min, 99%; 14 min, 99%; 14.5 min, 2%; and 20 min, 2% of B. The quantification of metabolites was performed using a Dionex UltiMate 3000 UHPLC system (Thermo Fisher Scientific, Waltham, MA, USA) connected to a SolariX 12T Fourier transform ion cyclotron resonance mass spectrometer (Bruker Daltonics, Billerica, MA, USA) in the electrospray ionization positive-ion mode. The two continuous accumulation of selected ions windows were adjusted by a quadrupole filter to 200–700, and 500–1500 Daltons. Qualitative and quantitative data processing was performed by CycloBranch version 2.0.19 and Bruker Data Analysis 5.0 software, respectively. The LOD for extracellular TafC and FC were 2.6, and 3.1 ng/mL, respectively. In the pellet, the LODs were at 14.9, and 9.6 ng/g. The lowest detectable amount of FC/HFC was 0.3 fg per a single *Aspergillus* conidium. The LOQ values are summarized in Supplementary Materials Table S1.

### 2.5. Statistical Analysis

In three biological replicates, the liquid fermentation media were sampled at 48, 52, or 24, 31, 48, 54, and 72 h for pellets and supernatants, respectively. Each sample was analyzed in three technical replicates by mass spectrometry, providing nine points for statistical analyses (3 × 3). The differences in the metabolite levels among *A. fumigatus* strains are presented as standard box plots with outliers plotted as individual points (Supplementary Materials Figures S1–S3). The box plots were built using MS Excel 2016. Kruskal–Wallis One-Way ANOVA with Bonferroni (All-Pairwise) Multiple Comparison and Friedman’s Q Rank Test were used to compare the intracellular and extracellular levels’ differences in VF and VI *A. fumigatus* strains. Friedman’s Q Rank Test was explicitly used to test how the strain and growth-phase time affected siderophore levels. The *p*-values indicated that the growing curve shapes were similar for all five strains, and the total amounts of siderophores secreted during fungal growth were not strain-dependent (Supplementary Materials Table S2). Statistical analysis was performed by NCSS 9 statistical software (NCSS, Kaysville, UT, USA). 

## 3. Results 

### 3.1. Pigment Secretion Is Observed by Virus-Infected but Not VF A. fumigatus 

Through curing the VI *A. fumigatus* UK AF293 isolate (18–95) and re-introducing AfuPmV-1 into VF isolates, one isogenic VF (18–42) and two VI lines (19–40 and 19–42) were generated [5]. The VI USA AF293 isolate (10–53) was also included in the study (Table 1). The two infected strains are subcultures of the reference strain 293, that have been preserved independently in the US and UK laboratories [6]. Cultivation on malt extract agar revealed no differences in coloration between the five strains. Conversely, in an iron-limited mineral liquid medium, the VF strain was the only one not secreting pigment while producing higher mycelial dry weight (Supplementary Materials Figure S4).

### 3.2. Monitoring Extracellular Secretion Kinetics Reveals Differential Secretion of Siderophores

**Figure 1:**
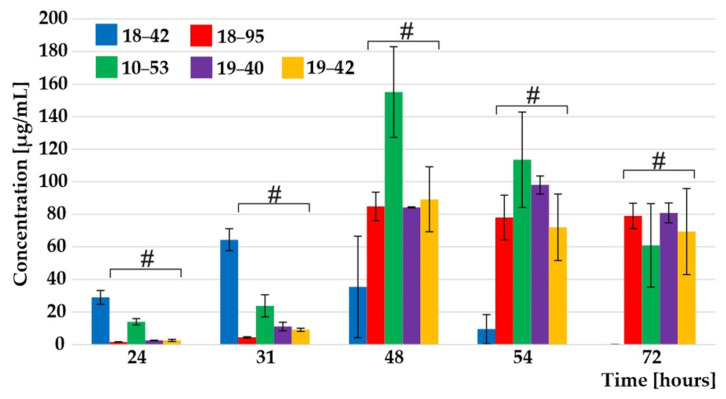
TafC time-related data in *A. fumigatus* supernatant. The VF *A. fumigatus* secretes TafC earlier than the VI strains, indicated by an # symbol. The error bars indicate the standard error of the mean, *n* = 9. See also Supplementary Materials Table S3.

The VF strain demonstrated the best fitness in iron restriction, represented by the fastest onset of exponential growth in an iron-limited mineral liquid medium. Quantification of the siderophore TafC, secreted in the supernatant and responsible for iron uptake, showed that the exponential phase and transitional production phase of the extracellular TafC were achieved at 24 and 31 h, respectively, and were significantly greater for the VF strain in contrast to the slower VI strains (Figure 1). Notably, study of a whole time course is important, rather than only selected time points: the data could have been interpreted differently if only selected time points had been used for reaching conclusions (see Figure 1 TafC secretion at 31 versus 72 h). The differences in the secretion rate of extracellular TafC between the VF and the VI strains were statistically significant (Kruskal–Wallis, *p* < 0.01) in the early and stationary growth phase (see Supplementary Materials Figure S1). 

FC is considered an ambiguous *A. fumigatus* siderophore in terms of cellular localization. In addition to its canonical intracellular and transcellular roles [8], reports indicate that a portion of the total FC can also be extracellular [9]. Indeed, substantial FC amounts were detected in culture supernatants of all five strains under study (Supplementary Materials Figure S2), although the total FC concentrations were an order of magnitude lower than those of TafC. The VF strain had higher FC concentrations, up to 3–4 µg/mL, at 24 and 31 h, as compared to the VI strains and this increase was statistically significant (Kruskal–Wallis, *p* < 0.01). 

In addition to the statistically significant differences between the VF and multiple VI strains, an important observation was made for 10–53 strain (Supplementary Materials Table S3). The fitness of 10–53 strain, characterized by its capacity to start the TafC secretion as early as possible, was the highest amongst all four VI strains (recalling that the re-infected strains were produced on the strain 18–95 parent). The enhanced fitness has been observed by 48 or 31 h of fungal growth for extracellular TafC or FC (*p* < 0.01), respectively. Similar observation could be made for intracellular FC and HFC in 48 h (Supplementary Materials Table S4).

### 3.3. Fungal Pellets but Not Conidia Have Different HFC/FC Ratios 

**Figure 2:**
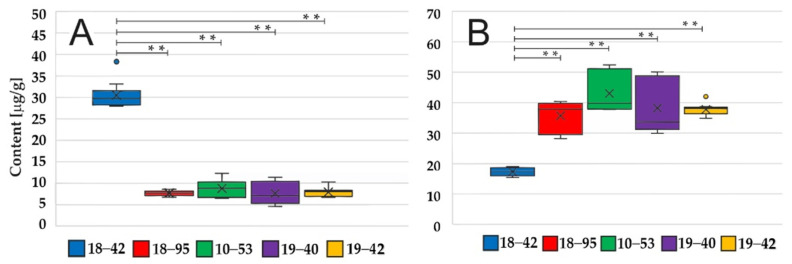
(**A**) Hydroxyferricrocin and (**B**) ferricrocin in *A. fumigatus* pellets. The VF *A. fumigatus* has higher HFC but lower FC content as compared to the VI strains at 52 h, *n* = 9. Asterisks (**) indicate statistical significance (Kruskal–Wallis, *p* < 0.01).

**Figure 3:**
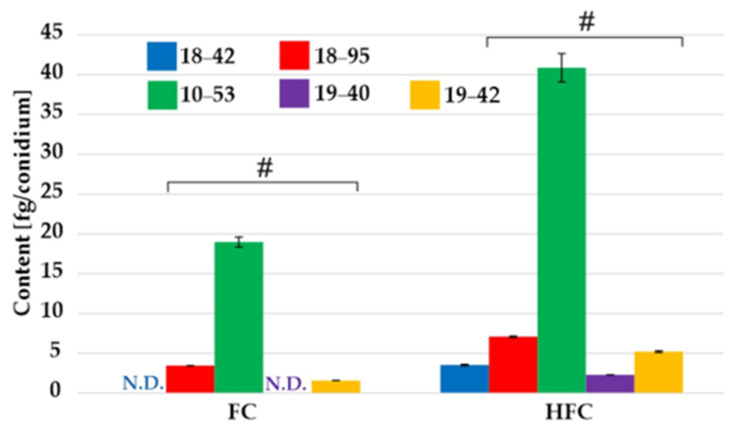
The HFC/FC content variation in *A. fumigatus* conidia. No significant differences or trends were observed between the VF and the VI strains, indicated by an # symbol. N.D. denotes the FC content below limit of detection, i.e., 0.3 fg/conidium. See also Supplementary Materials Table S4.

The HFC/FC content in the pellet was determined in the stationary phase, i.e., 48 and 52 h of cultivation, and the intracellular content was similar at both time points (Supplementary Materials Figure S3). The VF strain had higher HFC but lower FC content, respectively (Figure 2). These differences were statistically significant (Kruskal–Wallis, *p* < 0.01). Overall, the sum of both FC components (µg per g of a pellet) was similar in VF and VI strains (approximately 47 µg/g at 52 h). In agreement with the literature [10], HFC was preferentially stored in fungal conidia. The HFC and FC contents in the conidia of VF and VI strains were similar (Figure 3). 

## 4. Discussion

Mycoviruses have been reported to affect a range of host phenotypes, such as morphology, pigmentation, growth, virulence, pathogenicity, toxin production, and azole resistance [11]. Polymycovirus infection has been shown previously to enhance pigment production of a fungus [12], while other mycovirus infections in *A. fumigatus* may differentially affect host pigmentation [13]. Although *Aspergillus* spp. are capable of producing a variety of different pigments (aspergillin, asperenone, azafilones, and azanigerones A–F), *A. fumigatus* only produces melanin structures, which act as scavengers of reactive oxygen species and are putative factors of virulence [14]. Melanin structures or the precursors of either pyomelanin or dihydroxynaphthalene [15] were not detectable in our culture supernatants of VI strains from using matrix-assisted laser desorption mass spectrometry. Therefore, we concluded that the pigments secreted by the VI strains but absent in the culture of the VF strain were not associated with melanin production. Potentially, the color of the culture supernatant of the VI strains was a byproduct of the fungus processing the cultivation medium. However, since melanin structures are biopolymers difficult to analyze by mass spectrometry even following chemical or physicochemical cleavage, their presence cannot be completely excluded in VI strains. Of note, production of light absorbing, aromatic pigments may not be directly linked to *A. fumigatus* virulence [16]. Similarly, deletion of melanin biosynthesis was not always associated with significant fungal virulence in a murine model [15].

In an immunosuppressed mouse infection model, the closely related Aspergillus fumigatus polymycovirus 1M (AfuPmV-1M) infected *A. fumigatus* strain showed reduced mortality as compared to the VF strain. In that study, RNA sequencing and high-performance liquid chromatography (HPLC) analysis showed that the virus suppressed the expression of genes for gliotoxin synthesis and its production at the mycelial stage [17]. Another polymycovirus was shown to increase *A. fumigatus* virulence in the greater wax moth *Galleria mellonella* infection model [13]. To our knowledge, no direct link between mycovirus infection and siderophores has been reported previously; however, polymycoviruses were associated with carbon and nitrogen metabolism alterations [12]. In our study, the siderophore reservoir is consumed sooner by the VF *A. fumigatus* strain, which better withstood the competition for iron with *P. aeruginosa* [6]. The VF strain thus fares better with *P. aeruginosa* due to its altered iron-acquiring tactics. Additionally, the observed FC trend in the VF strain supports its better fitness, making it more resistant in iron competition against *P. aeruginosa*. At present, the role of HFC in *A. fumigatus* physiology and pathogenesis remains understudied and analytically; the position of the hydroxyl group in the FC structure has not yet been elucidated. Although a rather constant composition of the intracellular contents would be expected during the mycelial growth, future studies may expand our understanding of what the true ratio of HFC/FC should be.

Mycoviruses have been shown to modulate fungal fitness and virulence via a range of mechanisms. For example, some yeast mycoviruses encode killer toxins that kill sensitive cells through various mechanisms, such as inhibition of DNA replication, induction of membrane permeability changes, arrest of the cell cycle, or interfering with cell wall synthesis [18]. Mycovirus-encoded killer toxins or peptides have been demonstrated to control fungal infections, including aspergillosis in animals [19]. In *A. flavus*, mycovirus infection induced the production of toxigenic aflatoxins [20,21]. Similar results were obtained in partitivirus-transfected *A. ochraceus* strain overproducing ochratoxin A (OTA) [22]. Controversially, *AoOTApks1*, a polyketide synthase gene considered essential for ochratoxin production, was surprisingly absent in the genome of that OTA-producing isolate. In another report, *A. fumigatus* chrysovirus 41362 (AfuCV41362) suppressed the expression of several pathogenicity-associated host genes, including hypoxia adaptation and nitric oxide detoxification genes [23]. 

In our study, the viral proteins or RNA may interfere with non-ribosomal fungal siderophore synthesis and alter the fungal stress responses [6]. This could be achieved directly, via virus–host protein–protein interactions that disrupt host pathways, or, indirectly, via ‘off-target’ RNA silencing of host transcripts. AfuPmV-1 is a non-conventionally encapsidated mycovirus [4], whose double-stranded (ds) RNA genome is an easily accessible target for the antiviral machinery of the fungal host. Previous studies have shown that AfuPmV-1 silencing accounts for approximately 1/3 of small (s) RNAs present in virus-infected *A. fumigatus*, leading to differential expression of host genes [24]. Moreover, we speculate that virus gene expression in the infected fungal cell represents a substantial metabolic burden illustrated by a slower growth rate and lower dry cell weight achieved in the stationary phase, i.e., an effect similar to recombinant bacterial cells with high-copy-number plasmid DNA.

## 5. Conclusions

Mycoviruses may interfere with host-cell metabolism and, in so doing, alter production of fungal metabolites. As a result, viral infection may affect the outcome of bacterial–fungal competition in nature and patients. Our data suggest that the AfuPmV-1 proteins or RNA interfere with fungal siderophore synthesis and iron metabolism. Iron metabolism is a critical aspect of the competition between *A. fumigatus* and *P. aeruginosa* [25]. Fungal virulence attenuation through transfection of *Aspergillus* with mycoviruses represents a promising experimental approach analogous to antibacterial phage therapy. The molecular definition of the active viral principles, i.e., linking the chemical structures present in viral particles inhibitory to fungal protein targets, is important. The inhibitors may act in a way similar to phosphopantetheine transferase inhibitors, or inhibitors of siderophore synthetases, in experimental antifungal therapies [26].

## Figures and Tables

**Table 1:** The growth characteristics of VF and VI *A. fumigatus* strains (see text for details). ^$^ Number of conidia harvested from solid medium. ^#^ Pellet cell dry weight (cdw) obtained from the liquid medium (*n* = 3).

Strain	Designation	Conidia ^$^ (×10^8^)	Cdw (mg) ^#^
18–42 (VF)	UK Af293 cured from AfuPmV-1	7.03	55.3 ± 3.4
18–95	UK Af293 with AfuPmV-1	2.75	42.6 ± 3.9
10–53	USA Af293 with AfuPmV-1	2.30	42.1 ± 0.5
19–40	18–42 re-infected with AfuPmV-1	2.25	43.1 ± 3.3
19–42	18–42 re-infected with AfuPmV-1	1.84	42.5 ± 3.9

## Supplementary Materials

The following are available online at https://www.mdpi.com/2309-608X/7/7/497/s1. Figure S1: The variation in TafC concentrations. Figure S2: The variation in extracellular FC concentrations. Figure S3: The variation in FC and HFC contents. Figure S4: The pigmentation in VF and VI strains. Table S1: The instrument LODs and LOQs. Table S2: Testing the strain and growth phase time effects on siderophore levels. Table S3: Statistical differences among extracellular TafC and FC levels. Table S4: Statistical differences among intracellular FC and HFC levels. Excel: statistics.
