# Correction: Patil et al. Freeing *Aspergillus fumigatus* of Polymycovirus Infection Renders It More Resistant to Competition with *Pseudomonas aeruginosa* Due to Altered Iron-Acquiring Tactics. *J. Fungi* 2021, *7*, 497

**DOI:** 10.3390/jof8070691

**Published:** 2022-06-29

**Authors:** Rutuja H. Patil, Ioly Kotta-Loizou, Andrea Palyzová, Tomáš Pluháček, Robert H. A. Coutts, David A. Stevens, Vladimír Havlíček

**Affiliations:** 1 Institute of Microbiology of the Czech Academy of Sciences, Vídeňská 1083, 142 20 Prague, Czech Republic; rutuja.patil@biomed.cas.cz (R.H.P.); palyzova@biomed.cas.cz (A.P.); tomas.pluhacek@biomed.cas.cz (T.P.); vlhavlic@biomed.cas.cz (V.H.); 2 Department of Analytical Chemistry, Faculty of Science, Palacký University, 17. Listopadu 12, 771 46 Olomouc, Czech Republic; 3 Department of Life Sciences, Imperial College London, London SW7 2AZ, UK; i.kotta-loizou13@imperial.ac.uk; 4 Department of Clinical, Pharmaceutical and Biological Science, University of Hertfordshire, Hatfield AL10 9AB, UK; r.coutts@herts.ac.uk; 5 California Institute for Medical Research, 2260 Clove Dr., San Jose, CA 95128, USA; 6 Division of Infectious Diseases and Geographic Medicine, Stanford University School of Medicine, Stanford, CA 95128, USA

## Error in Text, including Table and Figures

In the original publication [1], an error was present throughout, as a strain was mislabeled.

The strain referred to in the article as “19–47” should have been referred to as “19–42”.

A correction has been made to: Section 2. Materials and Methods, subsection 2.1. Isolates, first paragraph; Section 3. Results, subsection 3.1. Pigment Secretion Is Observed by Virus-Infected but Not VF *A. fumigatus*, first paragraph; Figures 1–3 and Table 1.

The Figure 1 should be changed to: 


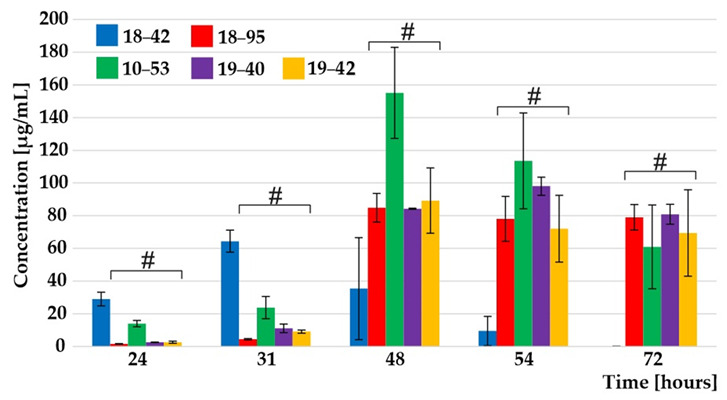


The Figure 2 should be changed to: 


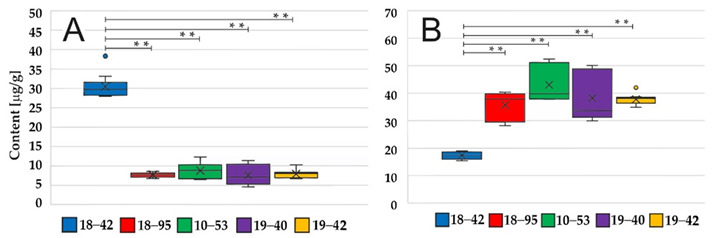


The Figure 3 should be changed to: 


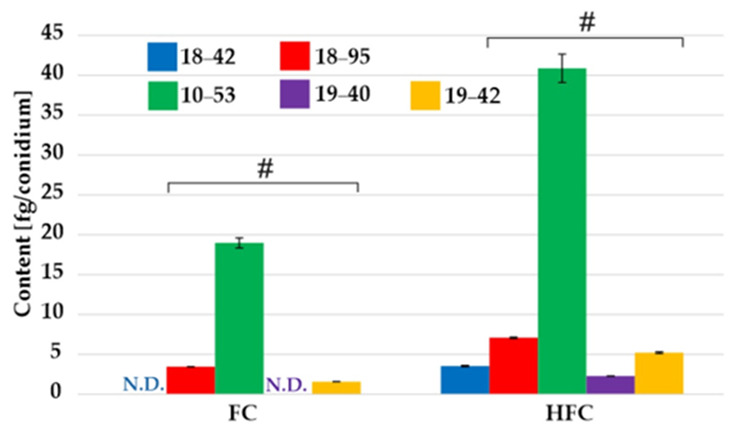


The Table 1 should be changed to:

**Table 1:** The growth characteristics of VF and VI *A. fumigatus* strains (see text for details). ^$^ Number of conidia harvested from solid medium. ^#^ Pellet cell dry weight (cdw) obtained from the liquid medium (*n* = 3).

Strain	Designation	Conidia ^$^ (×10^8^)	Cdw (mg) ^#^
18–42 (VF)	UK Af293 cured from AfuPmV-1	7.03	55.3 ± 3.4
18–95	UK Af293 with AfuPmV-1	2.75	42.6 ± 3.9
10–53	USA Af293 with AfuPmV-1	2.30	42.1 ± 0.5
19–40	18–42 re-infected with AfuPmV-1	2.25	43.1 ± 3.3
19–42	18–42 re-infected with AfuPmV-1	1.84	42.5 ± 3.9

The authors apologize for any inconvenience caused and state that the scientific conclusions have been unaffected. This correction was approved by the Academic Editor.

## References

[1] Patil R.H., Kotta-Loizou I., Palyzová A., Pluháček T., Coutts R.H.A., Stevens D.A., Havlíček V. (2021). Freeing *Aspergillus fumigatus* of Polymycovirus Infection Renders It More Resistant to Competition with *Pseudomonas aeruginosa* Due to Altered Iron-Acquiring Tactics. J. Fungi..

