# Supplementary material for: Freeing Aspergillus fumigatus of Polymycovirus Infection Renders It More Resistant to Competition with Pseudomonas aeruginosa Due to Altered Iron-Acquiring Tactics
Source: J Fungi (Basel). 2021 Jun 22;7(7):497. doi: 10.3390/jof7070497 (PMC8306778; doi:10.3390/jof7070497)
Supplement: Supplementary file 1 [file jof-07-00497-s001.zip › jof-1264329-supplementary-conv-done.pdf]

Journal of *Fungi*

Supplementary Material

Freeing *Aspergillus fumigatus* of Polymycovirus Infection Renders It more Resistant to Competition with *Pseudomonas aeruginosa* due to Altered Iron-Acquiring Tactics

Rutuja H. Patil [1,2], Ioly Kotta-Loizou [3], Andrea Palyzová [1], Tomáš Pluháček [1,2], Robert H. A. Coutts [4], David A. Stevens [5,6,*] and Vladimír Havlíček [1,2]

1 Institute of Microbiology of the Czech Academy of Sciences, Vídeňská 1083, 142 20 Prague, Czech Republic; rutuja.patil@biomed.cas.cz (R.H.P.); palyzova@biomed.cas.cz (A.P.); tomas.pluhacek@biomed.cas.cz (T.P.); vlhavlic@biomed.cas.cz (V.H.)
2 Department of Analytical Chemistry, Faculty of Science, Palacký University, 17. Listopadu 12, 771 46 Olomouc, Czech Republic
3 Department of Life Sciences, Imperial College London, London SW7 2AZ, UK; i.kotta-loizou13@imperial.ac.uk
4 Department of Clinical, Pharmaceutical and Biological Science, University of Hertfordshire, Hatfield AL10 9AB, UK; r.coutts@herts.ac.uk
5 California Institute for Medical Research, 2260 Clove Dr., San Jose, CA 95128, USA
6 Division of Infectious Diseases and Geographic Medicine, Stanford University School of Medicine, Stanford, CA 95128, USA
* Correspondence: stevens@stanford.edu

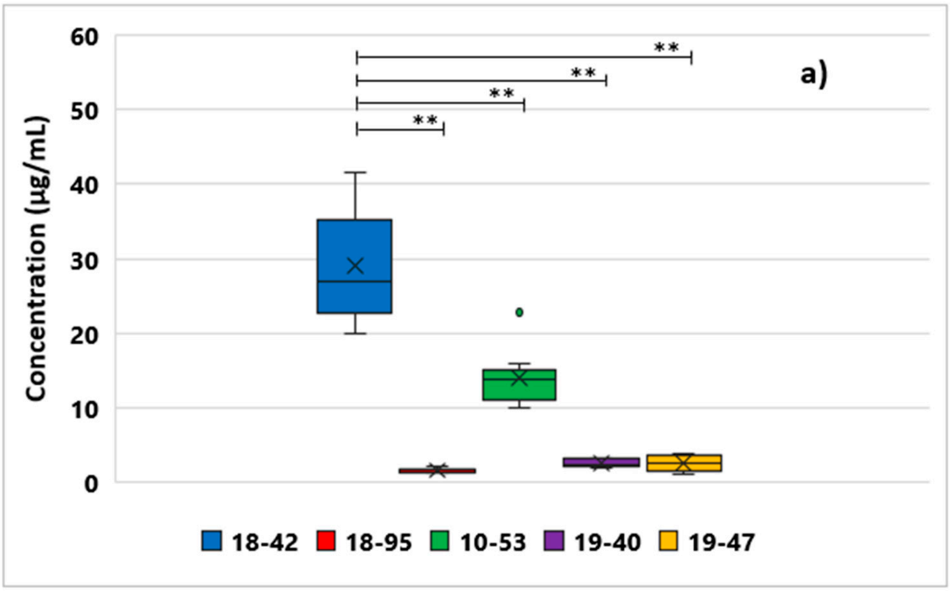

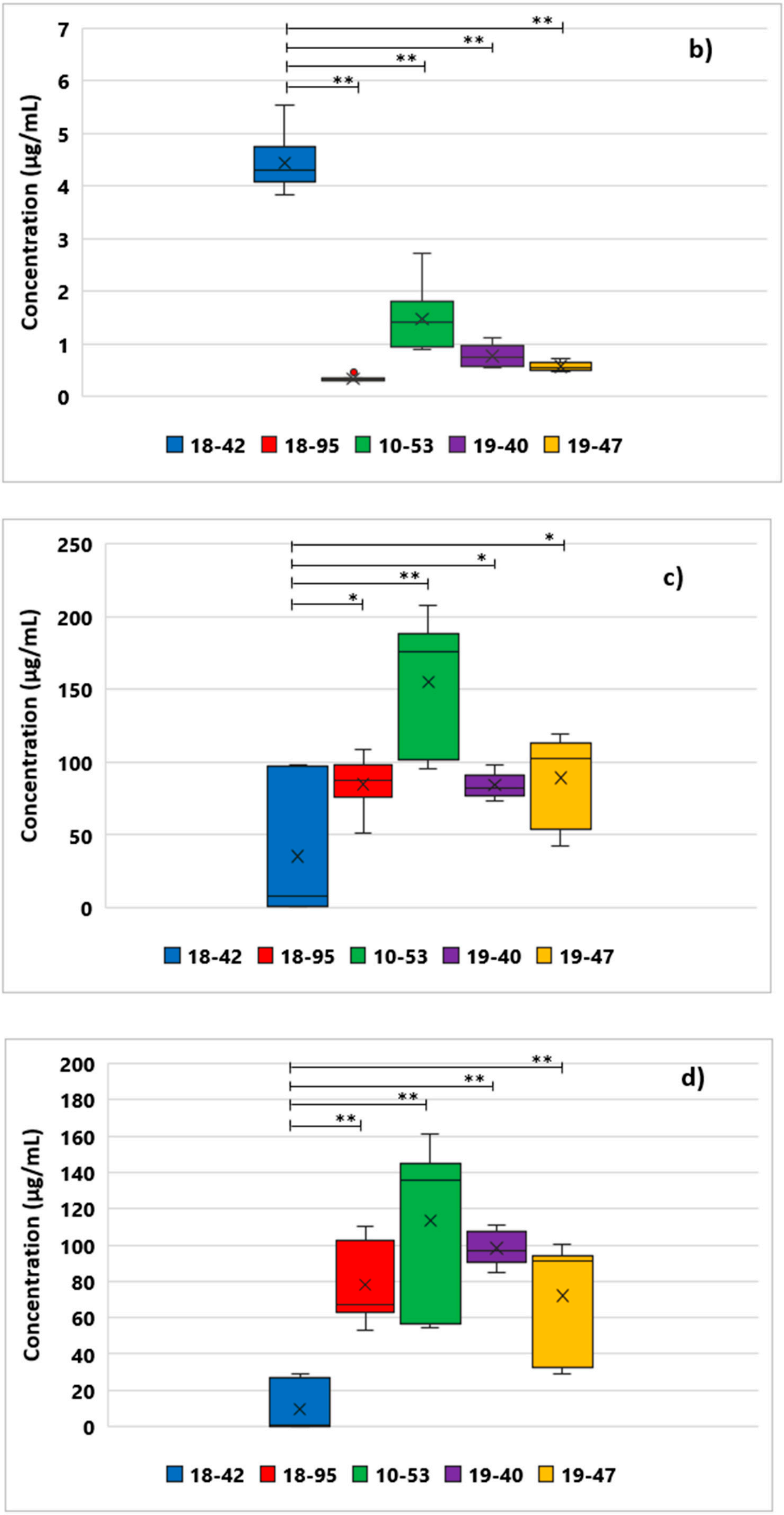
b)
Concentration (µg/mL)
7
6
5
4
3
2
1
0
**
**
**
**
18-42 18-95 10-53 19-40 19-47
c)
Concentration (µg/mL)
250
200
150
100
50
0
*
*
**
*
18-42 18-95 10-53 19-40 19-47
d)
Concentration (µg/mL)
200
180
160
140
120
100
80
60
40
20
0
**
**
**
**
18-42 18-95 10-53 19-40 19-47

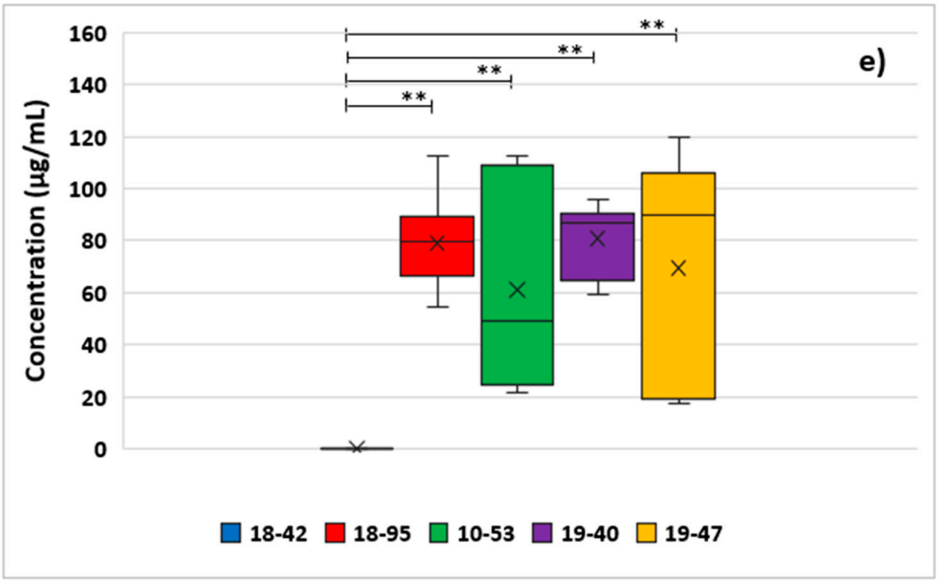

Figure S1. The variation in TafC concentrations for VF (18-42) and for non-VF (18-95, 10-53, 19-40, 19-47) *A. fumigatus* strains in fermentation medium collected in (**a**) 24 h, (**b**) 31 h, (**c**) 48 h, (**d**) 54 h, (**e**) 72 h**.** The Kruskal-Wallis One-Way ANOVA with Bonferroni Multiple Comparison results are represented by line segments marked by * ($P < 0.05$), * * ($P < 0.01$), $n = 9$.

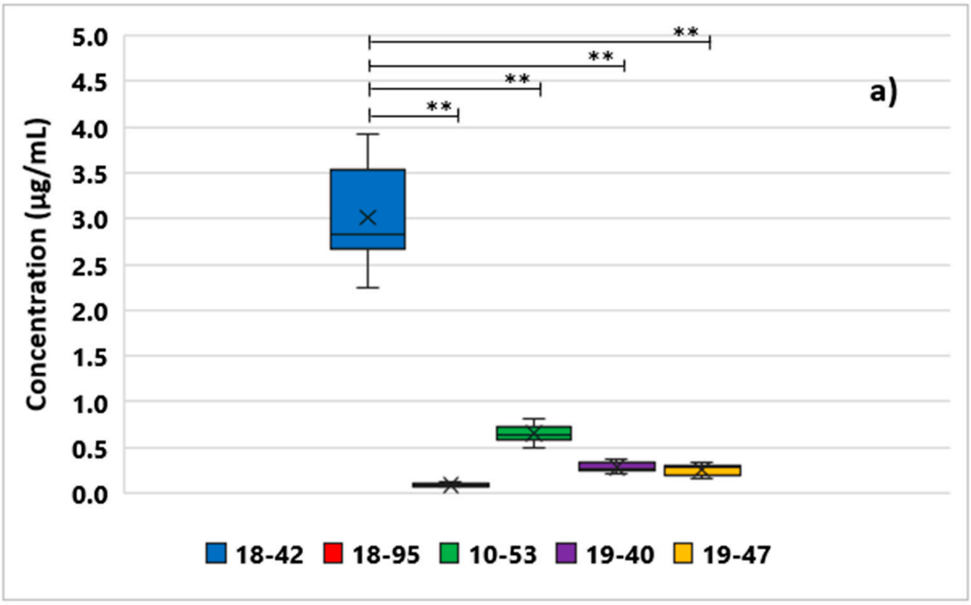

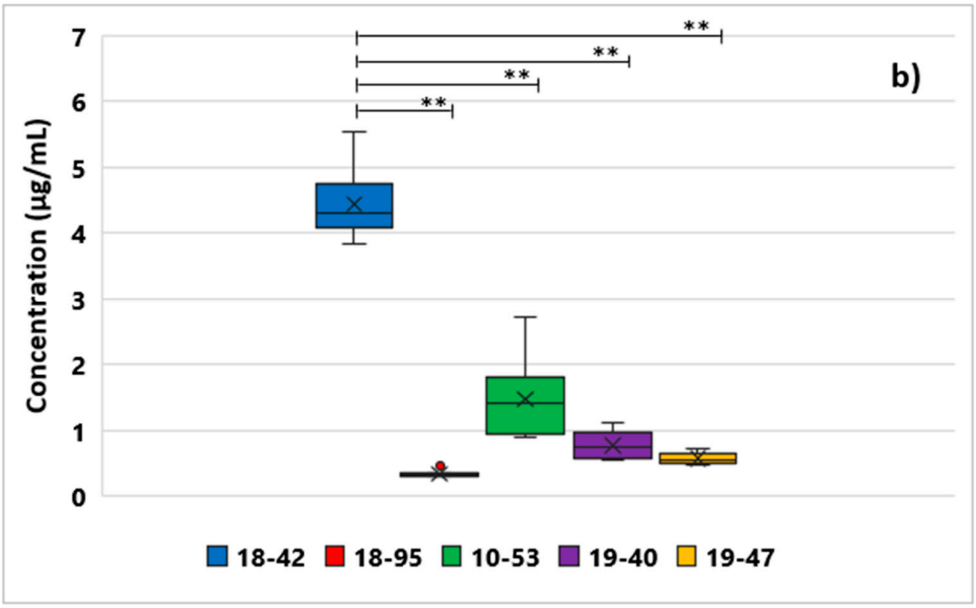
b)
Concentration (µg/mL)
0
1
2
3
4
5
6
7
**
**
**
**
18-42
18-95
10-53
19-40
19-47

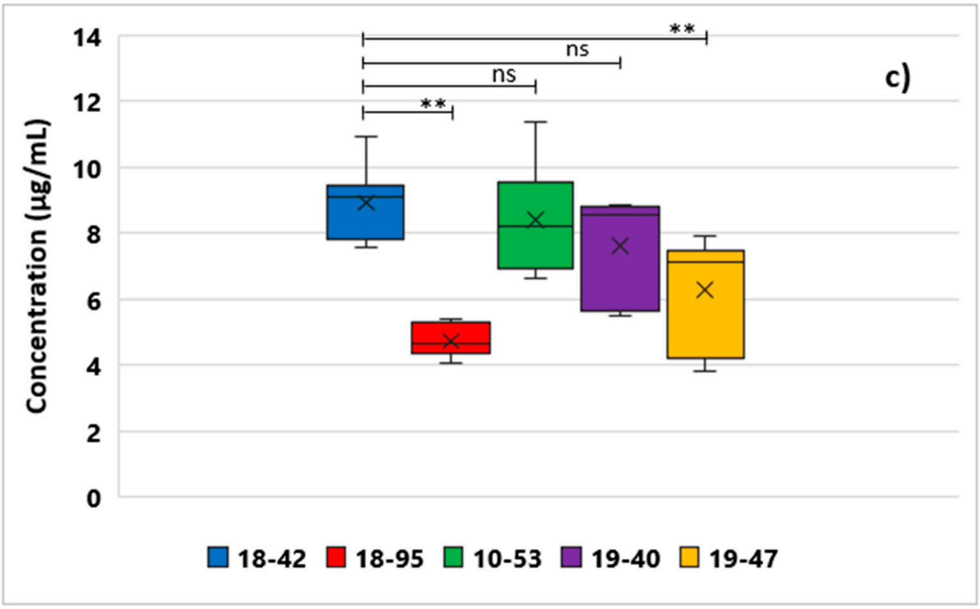
c)
Concentration (µg/mL)
0
2
4
6
8
10
12
14
**
ns
ns
**
18-42
18-95
10-53
19-40
19-47

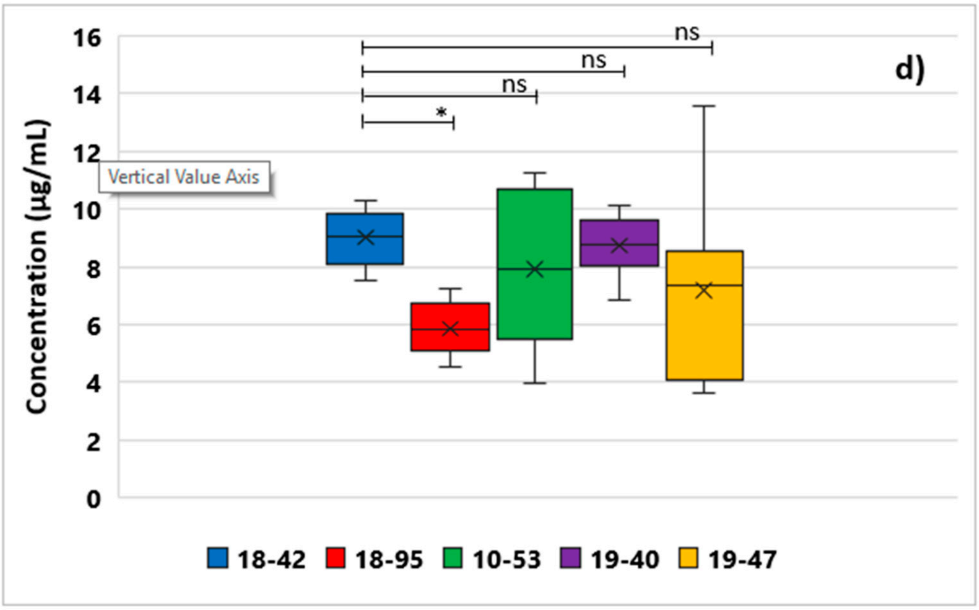

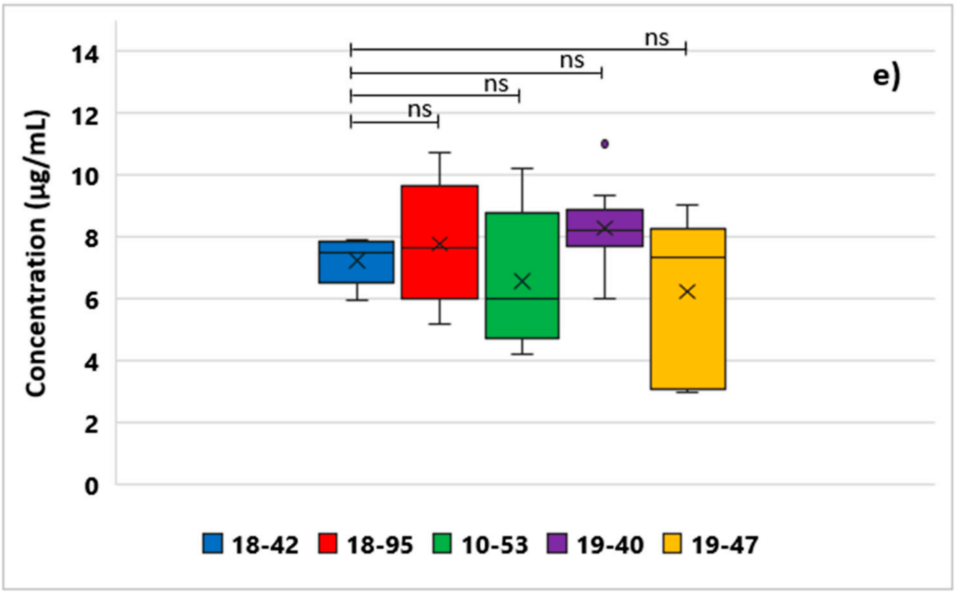

Figure S2. The variation in extracellular FC concentrations for VF (18-42) and for non-VF (18-95, 10-53, 19-40, 19-47) *A. fumigatus* strains in fermentation medium collected in (**a**) 24, (**b**) 31 h, (**c**) 48 h, (**d**) 54 h, (**e**) 72 h. The Kruskal-Wallis One-Way ANOVA with Bonferroni Multiple Comparison results are represented by line segments marked by * ($P < 0.05$), * * ($P < 0.01$) and ns (statistically insignificant), $n = 9$.

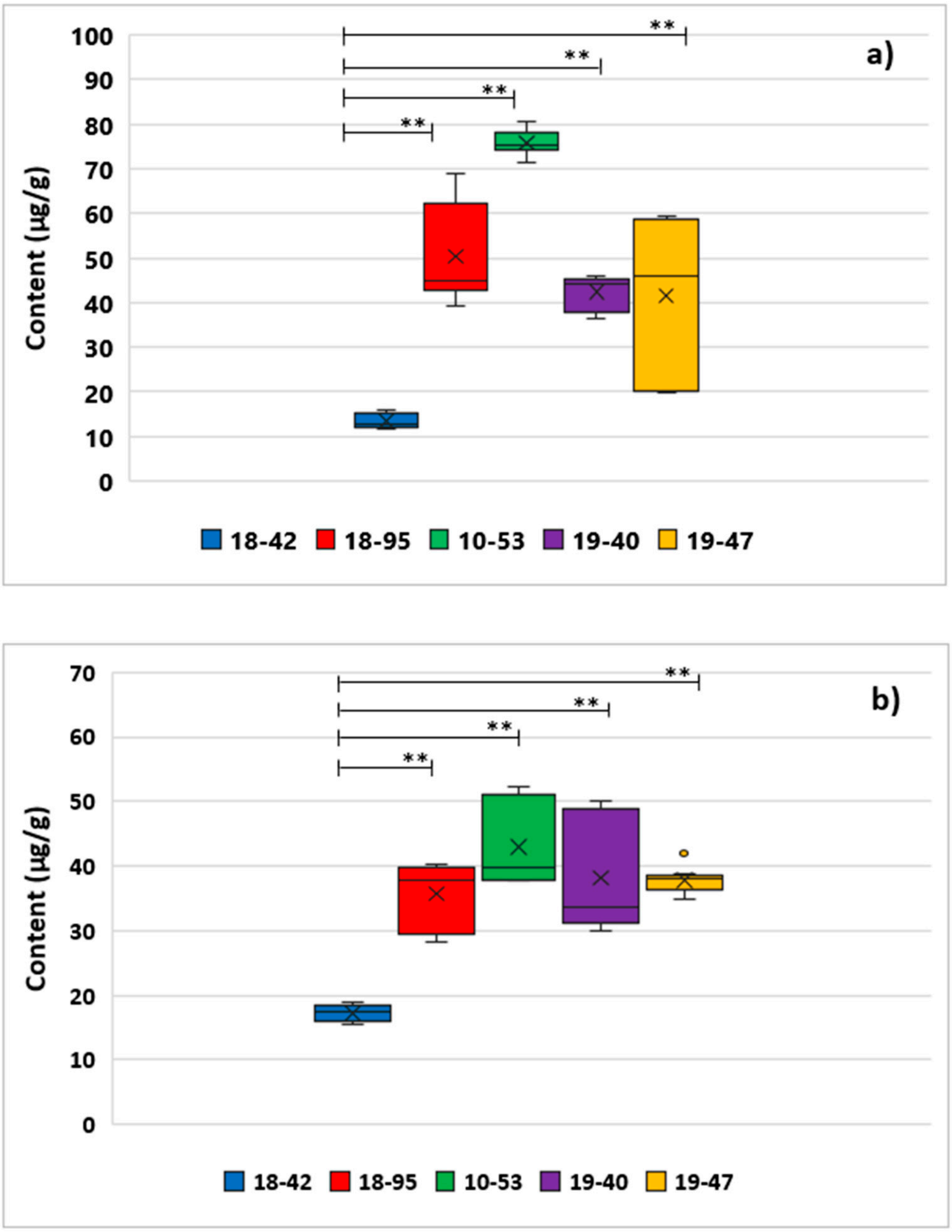
a)
Content (µg/g)
100
90
80
70
60
50
40
30
20
10
0
**
18-42
18-95
10-53
19-40
19-47
b)
Content (µg/g)
70
60
50
40
30
20
10
0
**
18-42
18-95
10-53
19-40
19-47

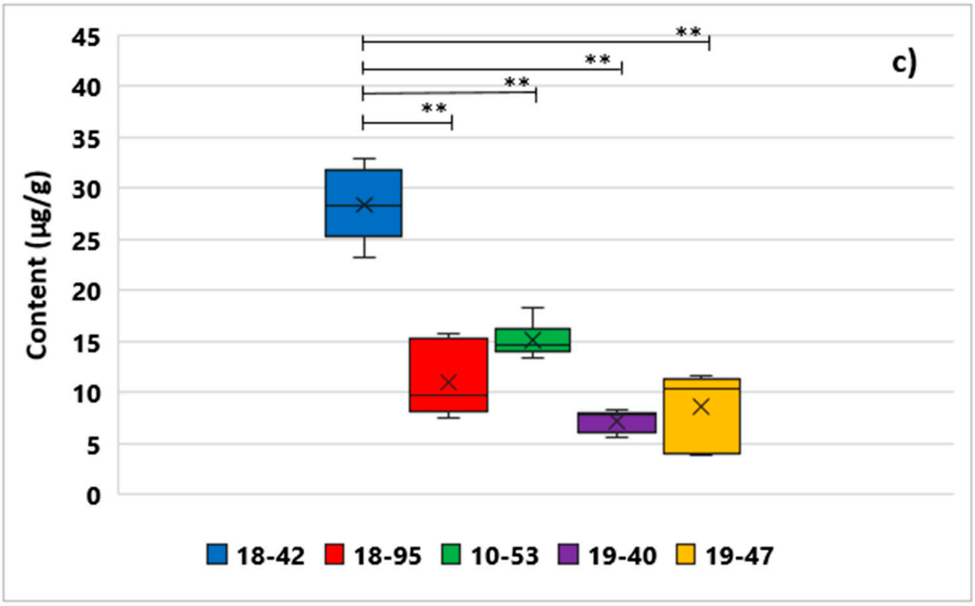

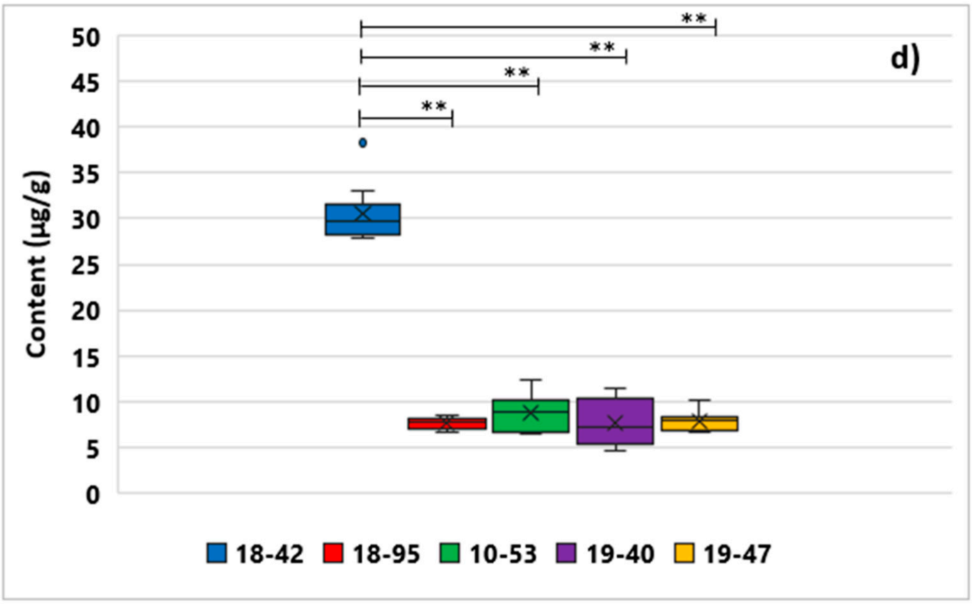

Figure S3. The variation in FC (a, b) and HFC (c,d) contents for VF (18-42) and for non-VF (18-95, 10-53, 19-40, 19-47) *A. fumigatus* strains in pellets collected in 48 h (**a,c**) and 52 h (**b,d**). The Kruskal-Wallis One-Way ANOVA with Bonferroni Multiple Comparison results are represented by line segments marked by * * ($P < 0.01$), $n = 9$. The similarity in HFC/FC content at both time points is derived from Friedman Q rank test (Supplementary Table S4, $P < 0.05$).

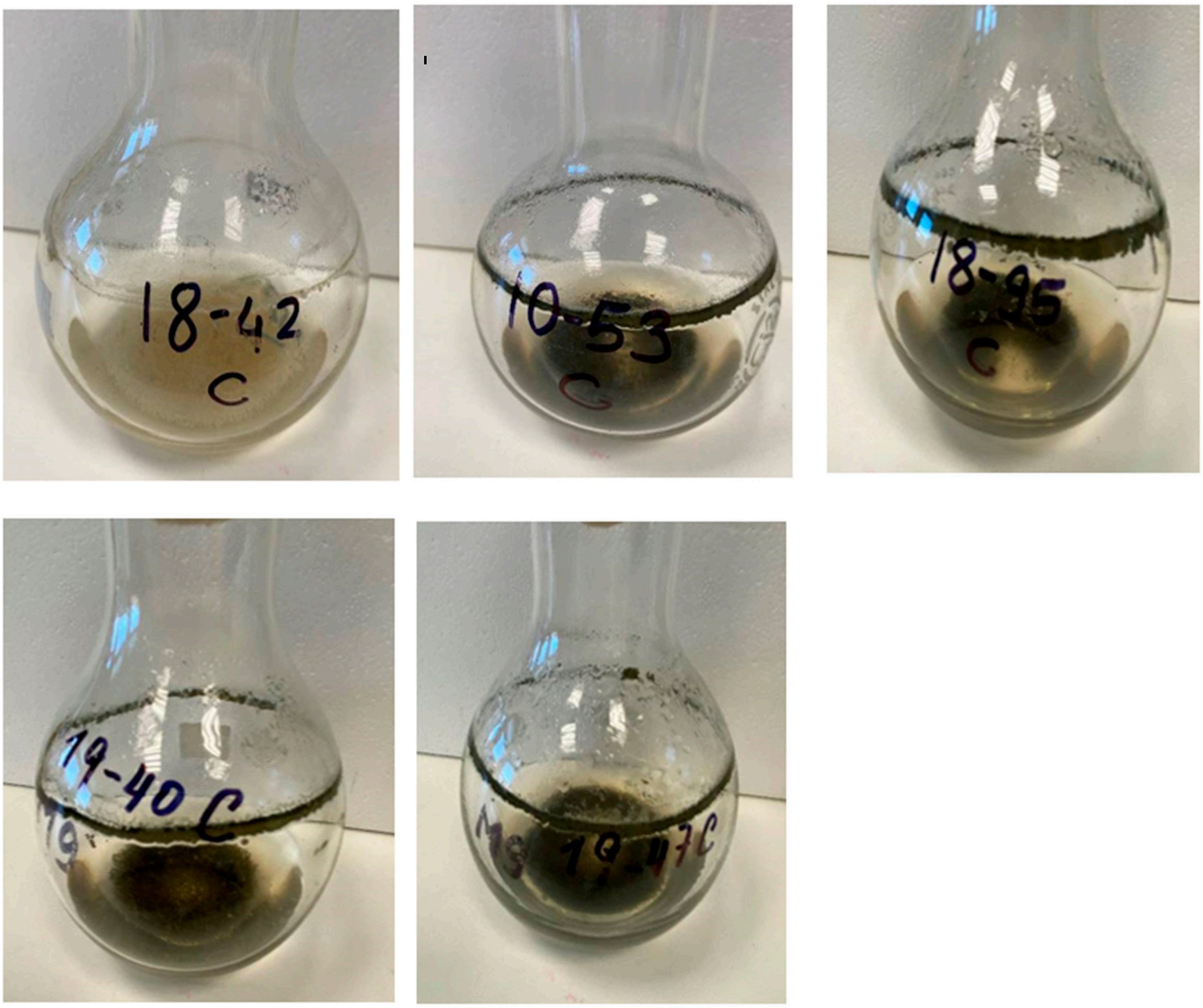

Figure S4. The pigmentation in VF and VI strains. *Aspergillus* strains (biological replicate "C") are displayed from the top to the right in the order: 18-42 (VF), 10-53 (VI), 18-95 (VI), 19-40 (VI), and 19-47 (VI).

Table S1. The instrument limits of detection (LODs) and limits of quantification (LOQs).

Analyte	Matrix	LOD	LOQ
TafC	Supernatant	2.6 ng/mL	8.7 ng/mL
FC	Supernatant	3.1 ng/mL	10.4 ng/mL
TafC	Pellet	14.9 ng/g	50.9 ng/g
FC	Pellet	9.6 ng/g	32.9 ng/g
FC/HFC	Conidia	0.3 fg/conidium	0.9 fg/conidium

Table S2: Testing the strain and growth phase time effects on siderophore levels. *P* values indicating staTable 0. Friedman's Q Rank Test). The non-statistically significant values returned for the intracellular FC, intracellular HFC and their sum indicated that the growing curve shapes were same for all five strains and the absolute amounts of the secreted siderophores were not strain dependent.

Siderophore	Friedman Q Rank Test
TafC (extracellular)	0.0228
FC (extracellular)	0.0018
FC (intracellular)	0.1797
HFC (intracellular)	0.6547
Sum of HFC and FC (intracellular)	0.1797

Table S3. Statistical differences among extracellular TafC and FC levels in VF and VI strains performed using Kruskal-Wallis One-Way ANOVA with Bonferroni (All-Pairwise) Multiple Comparison. * - *P*<0.05, ** - *P*<0.01, n.s. – not statistically significant.

	**24 h**	
**Strain**	**Extracellular TafC**	**Extracellular FC**
18-42	18-95**, 10-53**, 19-40**, 19-47**	18-95**, 10-53**, 19-40**, 19-47**
18-95	18-42**, 10-53**	18-42**, 10-53**
10-53	18-42**, 18-95**, 19-40**, 19-47**	18-42**, 18-95**, 19-40*, 19-47*
19-40	18-42**, 10-53**	18-42**, 10-53*
19-47	18-42**, 10-53**	18-42**, 10-53*
	**31 h**	
**Strain**	**Extracellular TafC**	**Extracellular FC**
18-42	18-95**, 10-53**, 19-40**, 19-47**	18-95**, 10-53**, 19-40**, 19-47**
18-95	18-42**, 10-53**	18-42**, 10-53**
10-53	18-42**, 18-95**, 19-40**, 19-47**	18-42**, 18-95**, 19-40**, 19-47**
19-40	18-42**, 10-53**	18-42**, 10-53**
19-47	18-42**, 10-53**	18-42**, 10-53**
	**48 h**	
**Strain**	**Extracellular TafC**	**Extracellular FC**
18-42	18-95*, 10-53**, 19-40*, 19-47*	18-95**, 19-47**
18-95	18-42*, 10-53**	18-42**, 10-53**,19-40**
10-53	18-42**, 18-95**, 19-40**, 19-47**	18-95**, 19-47*
19-40	18-42*, 10-53**	18-95**
19-47	18-42*, 10-53**	18-42**, 10-53*
	**54 h**	
**Strain**	**Extracellular TafC**	**Extracellular FC**
18-42	18-95**, 10-53**, 19-40**, 19-47**	18-95*
18-95	18-42**	18-42*, 19-40*

10-53	18-42**, 19-47*	n.s.
19-40	18-42**	18-95*
19-47	18-42**, 10-53*	n.s.
	**72 h**	
**Strain**	**Extracellular TafC**	**Extracellular FC**
18-42	18-95**, 10-53**, 19-40**, 19-47**	n.s.
18-95	18-42**	n.s.
10-53	18-42**	n.s.
19-40	18-42**	n.s.
19-47	18-42**	n.s.

Table S4. Statistical differences among intracellular FC and HFC levels in VF and VI strains performed using Kruskal-Wallis One-Way ANOVA with Bonferroni (All-Pairwise) Multiple Comparison. * - $P<0.05$, ** - $P<0.01$, n.s. – not statistically significant.

	**48 h**	
**Strain**	**Intracellular FC**	**Intracellular HFC**
18-42	18-95**, 10-53**, 19-40**, 19-47**	18-95**, 10-53**, 19-40**, 19-47**
18-95	18-42**, 10-53**	18-42**, 10-53*
10-53	18-42**, 18-95**, 19-40**, 19-47**	18-42**, 18-95*, 19-40**, 19-47**
19-40	18-42**, 10-53**	18-42**, 10-53**
19-47	18-42**, 10-53**	18-42**, 10-53**
	**52 h**	
**Strain**	**Intracellular FC**	**Intracellular HFC**
18-42	18-95**, 10-53**, 19-40**, 19-47**	18-95**, 10-53**, 19-40**, 19-47**
18-95	18-42**	18-42**
10-53	18-42**	18-42**
19-40	18-42**	18-42**
19-47	18-42**	18-42**
